# Morphological Spectrum and Pathological Parameters of Type 2 Endometrial Carcinoma: A Comparison With Type 1 Endometrial Cancers

**DOI:** 10.7759/cureus.11025

**Published:** 2020-10-18

**Authors:** Atif A Hashmi, Syeda N Iftikhar, Javaria Ali, Fatima Shaheen, Farhath Afroze, Abira Imran

**Affiliations:** 1 Pathology, Liaquat National Hospital and Medical College, Karachi, PAK; 2 Internal Medicine, MNR Medical College, Hyderabad, IND; 3 Internal Medicine, Deccan College of Medical Sciences, Hyderabad, IND; 4 Statistics, Liaquat National Hospital and Medical College, Karachi, PAK

**Keywords:** endometrial carcinoma, endometrioid carcinoma, serous carcinoma, clear cell carcinoma, carcinosarcoma, type 1 endometrial carcinoma, type 2 endometrial carcinoma

## Abstract

Introduction

Endometrial cancers (ECs) are the most common gynecological malignancies. Based on morphology and pathogenesis, ECs are segregated into type 1 and 2 ECs. Types 1 ECs are those tumors that are estrogen-driven, whereas type 2 ECs are more aggressive and are independent of hormonal status. In the proposed study, we evaluated the clinicopathological parameters of type 2 ECs and its comparison with type 1 ECs.

Methods

We retrospectively analyzed seven-year data from archives of pathology, Liaquat National Hospital, from January 2013 to December 2019. All patients underwent radical surgeries for diagnosed EC on endometrial biopsy. All specimens were of total abdominal hysterectomy with bilateral salpingo-oophorectomy, omentectomy, and peritoneal sampling, along with pelvic lymphadenectomy. Records regarding tumor type, grade, depth of myometrial invasion, and ovarian, omental, nodal, and parametrial involvement were assessed.

Results

A total of 129 cases of ECs were included in the study. The mean age of the patients was 57.6 ± 9.3 years. Majority of the cases were type 1 ECs (82.2%). The most common histological type of EC was endometrioid (82.2%) followed by serous carcinoma (10.1%). Most of the tumors were grade 1 (42.6%) and the International Federation of Gynecology and Obstetrics (FIGO) stage I (72.8%). Nodal metastases were present in eight cases (6.2%) and adnexal involvement was present in 12 cases (9.3%). We found a significant association of the type of EC with lymphovascular invasion, nodal metastasis, and adnexal involvement, whereas no significant association of EC type was seen with other clinicopathological characteristics.

Conclusions

Type 1 EC was the most frequent subtype of EC in our study. On the other hand, type 2 EC was significantly associated with nodal metastasis, lymphovascular invasion, and adnexal involvement, signifying the poor prognostic significance of this group of EC.

## Introduction

Endometrial cancers (ECs) are the most common gynecological malignancies. Based on morphology and pathogenesis, ECs are segregated into type 1 and 2 ECs. Types 1 ECs are those tumors that are estrogen-driven, while type 2 ECs are more aggressive and are independent of hormonal status [1,2]. Type 1 ECs include endometrioid and mucinous carcinoma, whereas type 2 ECs include serous, clear cell, and carcinosarcoma histologies. Phenotypically, type 2 cancers appear more atypical under a microscope, and, molecularly, they are distinguished based on early p53 mutations. Apart from histological types, other prognostic parameters of EC include depth of myometrial invasion, parametrial involvement, lymphovascular invasion, and ovarian and nodal metastasis. In addition, the role of various diagnostic and prognostic biomarkers has been studied in ECs [3,4]; however, a large review concluded that currently there is no routinely used biomarker for diagnostic or prognostic purposes in ECs [5].

The segregation of epithelial endometrial tumors into two groups (type 1 and 2) could help clinicians and oncologists determine the response of different treatment modalities in these two separate prognostic groups of EC. Recently, a molecular-based classification of ECs has been proposed; however, underdeveloped and resource-limited countries with lack of molecular diagnostic facilities continue to rely on conventional histological parameters. In the proposed study, we evaluated the clinicopathological parameters of type 2 EC and its comparison with type 1 EC.

## Materials and methods

We retrospectively analyzed seven-year data from archives of pathology, Liaquat National Hospital, from January 2013 to December 2019. All patients underwent radical surgeries for diagnosed EC on endometrial biopsy. All specimens were of total abdominal hysterectomy with bilateral salpingo-oophorectomy, omentectomy, and peritoneal sampling, along with pelvic lymphadenectomy. Records regarding tumor type, grade, depth of myometrial invasion, and ovarian, omental, nodal, and parametrial involvement were assessed.

Cases with unequivocal diagnosis of primary endometrial carcinoma were included in the study. Cases with post-neoadjuvant chemoradiation or secondary malignancies were excluded from the study. In addition, cases with non-epithelial (stromal) malignancies were also excluded from the study.

Gross examination of the specimens was performed according to standard protocols. Tumor dimension and extent of myometrial involvement were noted, and representative sections were submitted from the tumor. In cases where the tumor measured less than 2 cm, whole tumor was submitted. In cases of larger than 2-cm tumors, one section per centimeter of the tumor was submitted. In addition, representative sections from parametria, utero-cervical junction, cervix, fallopian tubes, ovaries, and the omentum were submitted. Pelvic lymph nodes were submitted entirely, and representative sections from the omentum were taken and submitted.

Immunohistochemical studies were conducted in selected cases of EC. Cytokeratin (CKAE1/3) immunostain was applied on all high-grade (grade 3) cases of EC to confirm epithelial nature of malignancy. In addition, p53 and napsin immunostains were performed for serous and clear cell carcinoma, respectively. In cases of suspicion of secondary malignancy, a panel of immunostains was applied, including CK7, CK20, TTF1, CDX2, GATA3, PAX8, and mamoglobin, to exclude metastatic carcinoma.

Data analysis was performed using Statistical Package for Social Sciences (SPSS) Version 26 (IBM Corp., Armonk, NY, USA). Chi-square test was used to check the association. P-values of ≤0.05 were considered as significant.

## Results

Descriptive characteristics of the population under study

**Figure 1:**
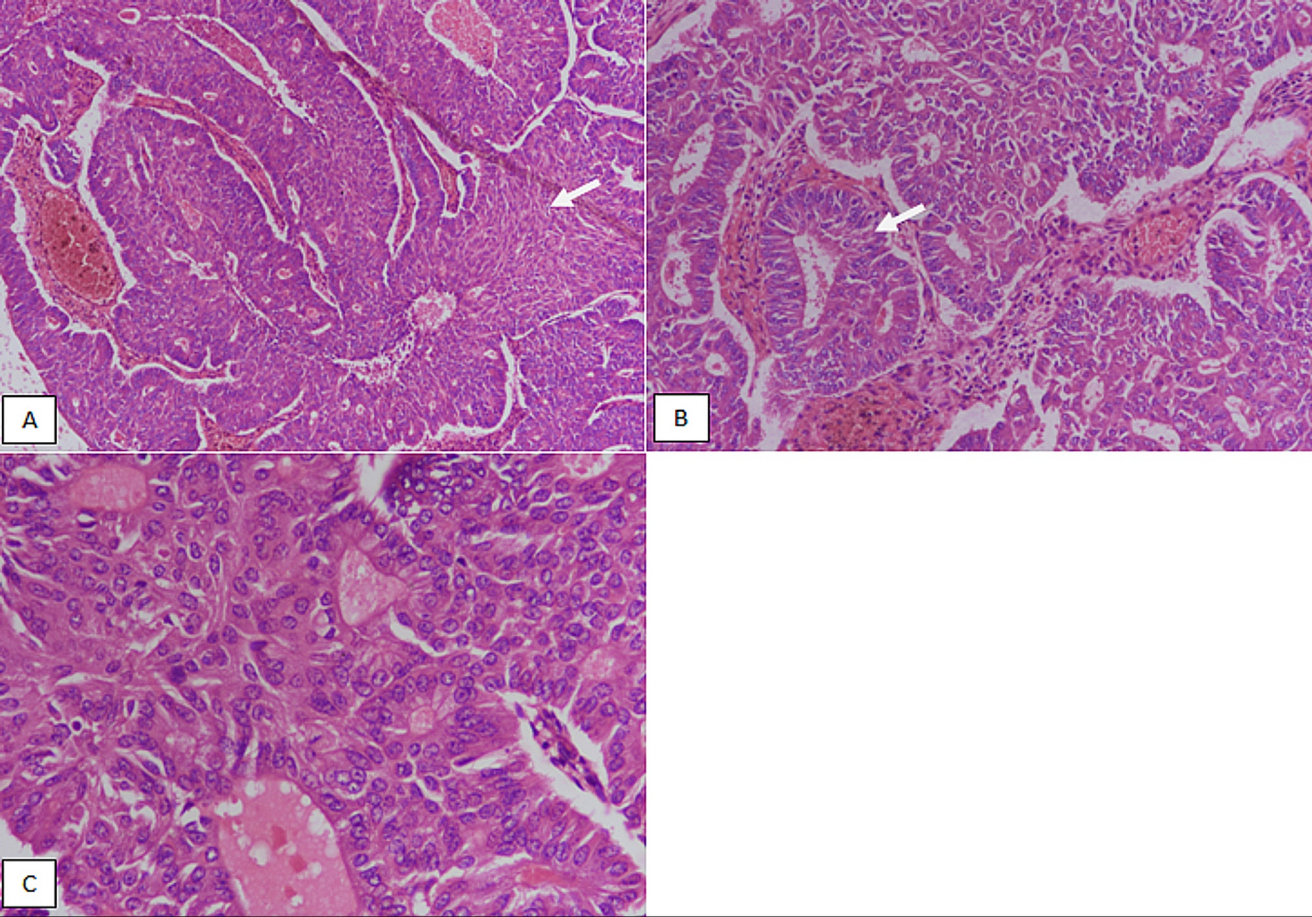
Type 1 endometrial carcinoma: endometrioid carcinoma of the endometrium. (A) H&E-stained sections at 100X magnification showing fused glandular configuration of tumor with focal solid area of growth (arrow). (B) 200X magnification showing gland formation (arrow). (C) 400X magnification showing low nuclear grade and absence of prominent nucleoli. H&E, hematoxylin and eosin

**Figure 2:**
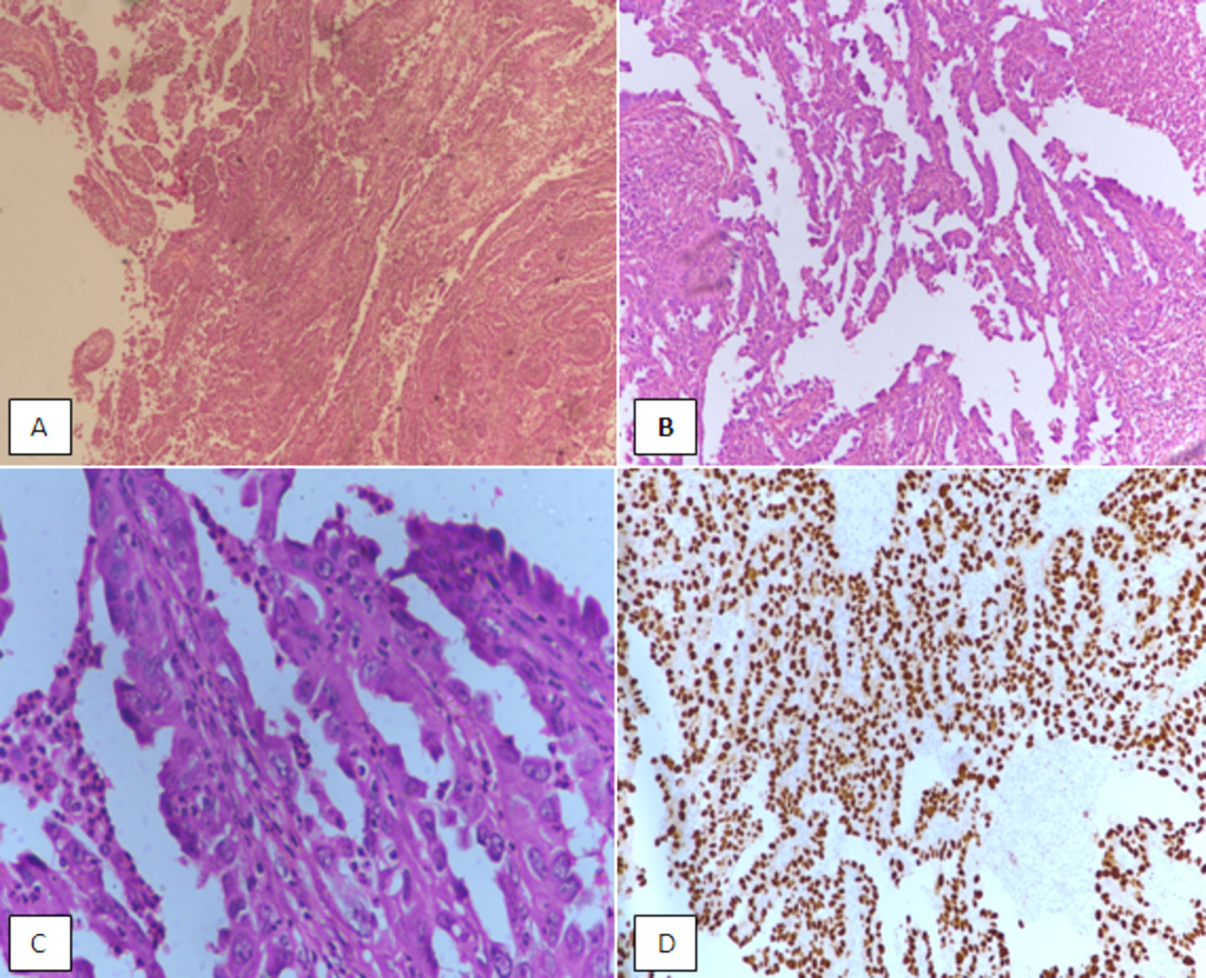
Type 2 endometrial carcinoma: serous carcinoma of the endometrium. (A) H&E sections at 40X magnification showing diffuse growth of tumor with vague papillary configuration. (B) 100X magnification showing papillary architecture. (C) 400X magnification showing atypical nuclei and prominent nucleoli. (D) p53 immunostain showing diffuse nuclear positivity in tumor cells. H&E, hematoxylin and eosin

**Figure 3:**
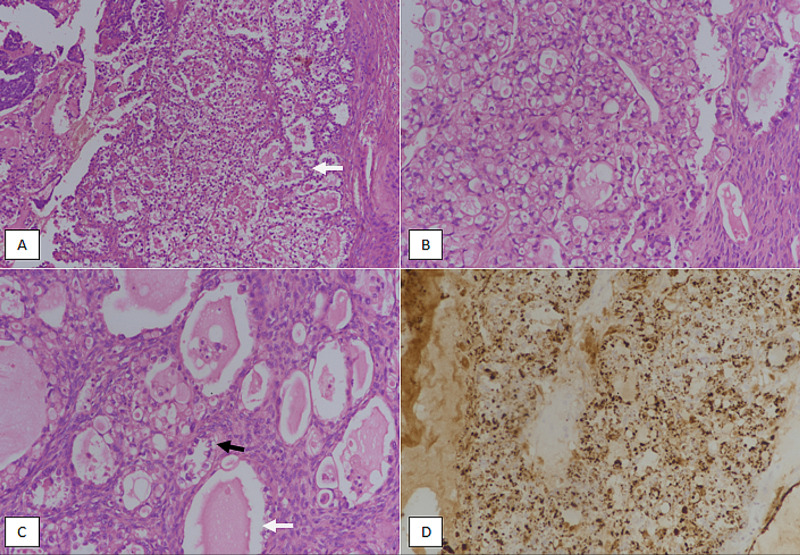
Type 2 endometrial carcinoma: clear cell carcinoma of the endometrium. (A) H&E sections at 100X magnification showing tumor glands lined by clear cells (white arrow). (B) 200X magnification depicting tumor with tubuloglandular architecture lined by clear cells with intraluminal secretions. (C) 400X magnification showing hobnailing of cells (black arrow) and intra-luminal secretions (white arrow). (D) Napsin A immunostain showing diffuse positivity in tumor cells. H&E, hematoxylin and eosin

**Figure 4:**
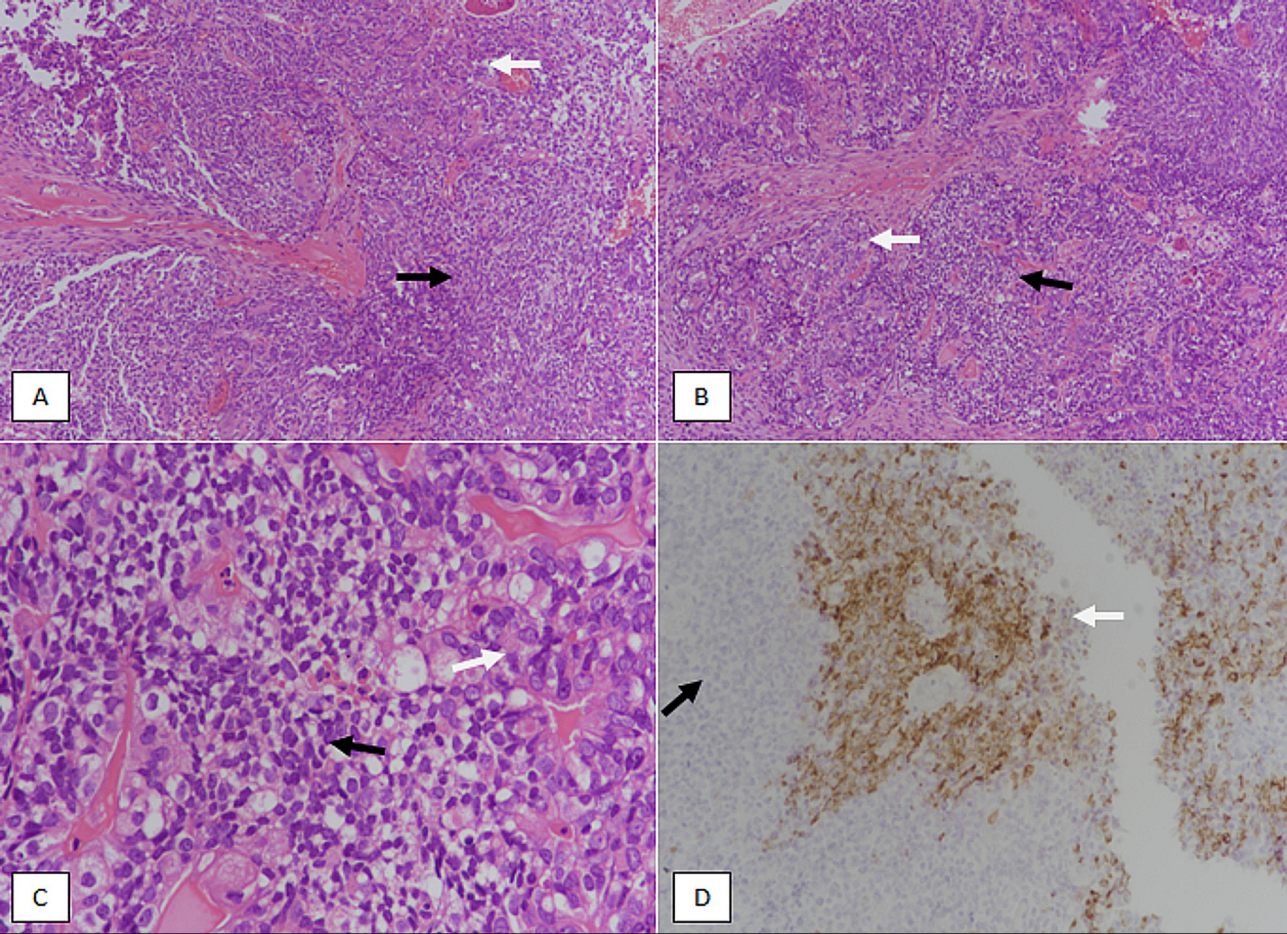
Type 2 endometrial carcinoma: carcinosarcoma of the endometrium. (A & B) H&E-stained sections at 100X magnification showing biphasic tumor with carcinomatous (white arrow) and sarcomatous components (black arrow). (C) 400X magnification showing carcinomatous component (white arrow) with highly atypical nuclei and sarcomatous component (black arrow). (D) Cytokeratin (CKAE1/3) immunostain showing positive staining in carcinomatous component (white arrow) and negative staining in the sarcomatous component (black arrow). H&E, hematoxylin and eosin

A total of 129 cases of EC were included in the study. The mean age of the patients was 57.6 ± 9.3 years. Most of the patients were over 50 years of age (77.5%) and postmenopausal (85.3%). Majority of the cases were of type 1 EC (82.2%). The most common histological type of EC was endometrioid (82.2%) followed by serous carcinoma (10.1%), carcinosarcoma (7%), and clear cell carcinoma (0.8%), as shown in Figures 1-4.

Most of the tumors were grade 1 (42.6%) and the International Federation of Gynecology and Obstetrics (FIGO) stage I (72.8%). Nodal metastases were present in 8 (6.2%) cases and adnexal involvement was present in 12 (9.3%) cases, as shown in Table 1.

**Table 1:** Descriptive statistics of population under study FIGO, International Federation of Gynecology and Obstetrics; T, tumor; N, nodal

Clinicopathological characteristics	Frequency (%)
Age group	
≤50 years	29 (22.5)
>50 years	100 (77.5)
Menopausal status	
Premenopausal	19 (14.7)
Postmenopausal	110 (85.3)
Histological type	
Endometrioid	106 (82.2)
Serous	13 (10.1)
Carcinosarcoma	9 (7.0)
Clear cell	1 (0.8)
Grade	
1	55 (42.6)
2	42 (32.6)
3	32 (24.8)
FIGO stage	
IA	51 (39.5)
IB	43 (33.3)
II	20 (15.5)
IIIA	8 (6.2)
IIIB	3 (2.3)
IV	4 (3.1)
T stage	
T1	92 (71.3)
T2	21 (16.3)
T3	12 (9.3)
T4	4 (3.1)
Lymphovascular invasion	
Present	10 (7.8)
Absent	119 (92.2)
N stage	
N0	121 (93.8)
N1	7 (5.4)
N2	1 (0.8)
Myometrial invasion	
Limited to the endometrium	6 (4.7)
More than half of the myometrium	71 (55)
Less than half of the myometrium	52 (40.3)
Cervical invasion	
Present	31 (24)
Absent	98 (76)
Adnexal involvement	
Present	12 (9.3)
Absent	117 (90.7)
Tumor type	
Type 1	106 (82.2)
Type 2	23 (17.8)

Association of the type of endometrial carcinoma with clinicopathological characteristics

We found a significant association of the type of EC with lymphovascular invasion, nodal metastasis, and adnexal involvement, whereas no significant association of EC type was seen with other clinicopathological characteristics. Although 21.7% of type 2 ECs were noted to have T stage T3/T4 compared to 10.4% of type 1 ECs, the difference was not statistically significant (Table 2).

**Table 2:** Association of clinicopathological parameters with type of endometrial carcinoma Chi-square test was applied. *p-Value significant as ≤0.05. FIGO, International Federation of Gynecology and Obstetrics; T, tumor; N, nodal

Clinicopathological characteristics	Type of endometrial carcinoma		p-Value
	Type 1	Type 2	
Age group			
≤50 years	26 (24.5)	3 (13)	0.283
>50 years	80 (75.5)	20 (87)	
Menopausal status			
Premenopausal	17 (16)	2 (8.7)	0.523
Postmenopausal	89 (84)	21 (91.3)	
FIGO stage			
IA	45 (42.5)	6 (26.1)	0.290
IB	34 (32.1)	9 (39.1)	
II	16 (15.1)	4 (17.4)	
IIIA	7 (6.6)	1 (4.3)	
IIIB	2 (1.9)	1 (4.3)	
IV	2 (1.9)	2 (8.7)	
T stage			
T1	78 (73.6)	14 (60.9)	0.223
T2	17 (16)	4 (17.4)	
T3	9 (8.5)	3 (13)	
T4	2 (1.9)	2 (8.7)	
Lymphovascular invasion			
Present	4 (3.8)	6 (26.1)	0.002*
Absent	102 (96.2)	17 (73.9)	
N stage			
N0	104 (98.1)	17 (73.9)	<0.001*
N1	1 (0.9)	6 (26.1)	
N2	1 (0.9)	0 (0)	
Myometrial invasion			
Limited to the endometrium	6 (5.7)	0 (0)	0.731
More than half of the myometrium	58 (54.7)	13 (56.5)	
Less than half of the myometrium	42 (39.6)	10 (43.5)	
Cervical invasion			
Present	26 (24.5)	5 (21.7)	0.777
Absent	80 (75.5)	18 (78.3)	
Adnexal involvement			
Present	7 (6.6)	5 (21.7)	0.023*
Absent	99 (93.4)	18 (78.3)	

Similarly, serous carcinoma and carcinosarcoma were noted to have a significantly higher association with nodal metastasis and lymphovascular invasion compared to endometrioid carcinoma (Table 3).

**Table 3:** Association of clinicopathological parameters with histological type Chi-square test was applied. *p-Value significant as ≤0.05. FIGO, International Federation of Gynecology and Obstetrics; T, tumor; N, nodal

Clinicopathological characteristics	Histological type				p-Value
	Endometrioid	Serous	Carcinosarcoma	Clear cell	
Age group					
≤50 years	26 (24.5)	3 (23.1)	0 (0)	0 (0)	0.425
>50 years	80 (75.5)	10 (76.9)	9 (100)	1 (100)	
Menopausal status					
Premenopausal	17 (16)	2 (15.4)	0 (0)	0 (0)	0.608
Postmenopausal	89 (84)	11 (84.6)	9 (100)	1 (100)	
FIGO stage					
IA	45 (42.5)	1 (7.7)	4 (44.4)	1 (100)	0.198
IB	34 (32.1)	6 (46.2)	3 (33.3)	0 (0)	
II	16 (15.1)	3 (23.1)	1 (11.1)	0 (0)	
IIIA	7 (6.6)	1 (7.7)	0 (0)	0 (0)	
IIIB	2 (1.9)	1 (7.7)	0 (0)	0 (0)	
IV	2 (1.9)	1 (7.7)	1 (11.1)	0 (0)	
T stage					
T1	78 (73.6)	7 (53.8)	6 (66.7)	1 (100)	0.383
T2	17 (16)	3 (23.1)	1 (11.1)	0 (0)	
T3	9 (8.5)	2 (15.4)	1 (11.1)	0 (0)	
T4	2 (1.9)	1 (7.7)	1 (11.1)	0 (0)	
Lymphovascular invasion					
Present	4 (3.8)	4 (30.8)	2 (22.2)	0 (0)	0.003*
Absent	102 (96.2)	9 (69.2)	7 (77.8)	1 (100)	
N stage					
N0	104 (98.1)	8 (61.5)	8 (88.9)	1 (100)	<0.001*
N1	1 (0.9)	5 (38.5)	1 (11.1)	0 (0)	
N2	1 (0.9)	0 (0)	0 (0)	0 (0)	
Myometrial invasion					
Limited to the endometrium	6 (5.7)	0 (0)	0 (0)	0 (0)	0.708
More than half of the myometrium	58 (54.7)	9 (69.2)	4 (44.4)	0 (0)	
Less than half of the myometrium	42 (39.6)	4 (30.8)	5 (55.6)	1 (100)	
Cervical invasion					
Present	26 (24.5)	4 (30.8)	1 (11.1)	0 (0)	0.698
Absent	80 (75.5)	9 (69.2)	8 (88.8)	1 (100)	
Adnexal involvement					
Present	7 (6.6)	3 (2.31)	2 (22.2)	0 (0)	0.071
Absent	99 (9.34)	10 (76.9)	7 (77.8)	1 (100)	

## Discussion

In this study, we evaluated the clinicopathological characteristics of different types of EC. We found that type 1 EC was more frequent compared to type 2 EC. Type 2 EC was noted to have a significantly higher association with lymphovascular invasion, nodal metastasis, and adnexal involvement.

Type 2 EC is considered more aggressive compared to type 1 cancer; however, these are less frequent. A study from India involving 84 cases of EC revealed that 11.9% were type 2 ECs, with more than half showing myometrial invasion [6]. In our study, type 2 EC comprised 17.8% of cases, and myometrial invasion was seen in all cases. Moreover, 56.5% cases depicted more than half of myometrial invasion, and adnexal involvement was seen in 21.7% cases. In our study, serous carcinoma was the most common type of type 2 EC followed by carcinosarcoma.

In this study, we noted that the majority of the patients were over 50 years of age and postmenopausal. Early age EC, especially in the presence of family history or synchronous/metachronous cancers, raises concerns about the microsatellite instability pathway induced EC [7].

We used the three-tiered FIGO grading system to grade type 1 EC in our study, and 51.9% cases were grade 1. Recently, FIGO recommended a binary scheme to grade endometrioid carcinoma, according to which grade 1 and grade 2 tumors are now known as low grade, and grade 3 tumors are defined as high grade [8]. According to this new classification, 48.1% of endometrioid carcinomas in our study were high grade.

A study conducted in Egypt compared the histological features of type 1 and 2 ECs and concluded that nuclear grade, mitotic activity, and low apoptotic count are features that predict type 2 EC histologically. In their investigation, 73.1% cases were type 1 ECs [9]. In our study, 82.2% cases were type 1 ECs. Song et al. studied 59 cases of stage I and II ECs, among which 83% were stage I, whereas 39% were grade 1 [10]. We found that 42.6% were grade 1 EC and 72.8% were FIGO stage I. A 10-year comparative study including 266 cases of type 2 EC revealed a significant association of type 2 EC with higher tumor dimension, the depth of myometrial invasion, tumor stage, and disease recurrence [11]. We found a significant association of type 2 EC with nodal metastasis, lymphovascular invasion and adnexal involvement; however, the association with tumor stage was not significant in our study. This lack of significance may be explained by the small number of type 2 EC cases in our study.

Lymphovascular invasion is one of the important pathways of tumor spread. We found that type 2 EC had a significantly higher rate of lymphovascular invasion compared to type 1 EC. A unique pathway of tumor spread in endometrial malignancies is transtubal spread, by which shed tumor cells from the endometrial cavity travel through the fallopian tubes to seed the pelvic cavity and involve the adnexa, even in the absence of significant myometrial invasion. In our study, adnexal involvement was noted in 21.7% of type 2 EC, which is alarming.

One of the major limitations of the study was the small sample size, and in particular the number of cases of type 2 EC was low. Second, the study design was retrospective and there was a lack of clinical follow-up. Large-scale prospective studies of type 2 EC are needed to evaluate cancer-specific survival in type 2 EC in our population. Moreover, recently a molecular-based classification of EC is proposed where four genomic subtypes of EC were identified and it was recommended that EC should be classified into the following categories: (1) p53 wild-type/copy number low, (2) p53 abnormal/copy number high, (3) polymerase E mutant, and (4) mismatch repair deficient [8]. Therefore, we recommended that prospective studies looking at the prognostic significance of this genomic classification should be conducted in our population.

## Conclusions

We found a low frequency of type 2 EC in our study, which is considered an aggressive variant of EC. We also found that type 2 ECs were associated with a higher frequency of nodal metastasis, which is one of the most important prognostic parameters of ECs. Similarly, lymphovascular invasion that is an indicator of regional and systemic metastasis was also noted more frequently in type 2 ECs along with adnexal involvement indicating poor prognostic profile of type 2 ECs.
